# Combination of Zinc Hyaluronate and Metronidazole in a Lipid-Based Drug Delivery System for the Treatment of Periodontitis

**DOI:** 10.3390/pharmaceutics11030142

**Published:** 2019-03-25

**Authors:** Attila Léber, Mária Budai-Szűcs, Edit Urbán, Péter Vályi, Attila Gácsi, Szilvia Berkó, Anita Kovács, Erzsébet Csányi

**Affiliations:** 1 Institute of Pharmaceutical Technology and Regulatory Affairs, Faculty of Pharmacy, University of Szeged, Szeged 6720, Hungary; leber.attila@pharm.u-szeged.hu (A.L.); maria.szucs@pharm.u-szeged.hu (M.B.-S.); gacsi.attila@pharm.u-szeged.hu (A.G.); berkosz@pharm.u-szeged.hu (S.B.); anita.kovacs@pharm.u-szeged.hu (A.K.); 2 Institute of Clinical Microbiology, Faculty of Medicine, University of Szeged, Szeged 6720, Hungary; tidenabru@freemail.hu; 3 Department of Periodontology, Faculty of Dentistry, University of Szeged, Szeged 6720, Hungary; valyi.peter@stoma.u-szeged.hu

**Keywords:** periodontitis, lipid drug delivery, zinc hyaluronate, amoxicillin, metronidazole

## Abstract

Background: Despite being a highly prevalent disease and a possible contributor to adult tooth loss, periodontitis possesses no well-established therapy. The aim of the recent study was the development and evaluation of a mucoadhesive monophase lipid formulation for the sustained local delivery of amoxicillin, metronidazole, and/or zinc hyaluronate or gluconate. Methods: To investigate our formulations, differential scanning calorimetry, X-ray diffraction, swelling, erosion, mucoadhesivity, drug release, and antimicrobial measurements were performed. Results: Differential scanning calorimetry (DSC) and X-ray diffraction (XRD) results show that the loaded drugs are in a suspended form, the softening of the formulations starts at body temperature, but a part remains solid, providing sustained release. Swelling of the lipid compositions is affected by the hydrophilic components, their concentration, and the strength of the coherent lipid structure, while their erosion is impacted by the emulsification of melted lipid components. Conclusions: Results of drug release and antimicrobial effectiveness measurements show that a sustained release may be obtained. Amoxicillin had higher effectiveness against oral pathogens than metronidazole or zinc hyaluronate alone, but the combination of the two latter could provide similar effectiveness to amoxicillin. The applied mucoadhesive polymer may affect adhesivity, drug release through the swelling mechanism, and antimicrobial effect as well.

## 1. Introduction

The plaque-induced forms of periodontal diseases are the most prevalent chronic inflammatory conditions seen in humans worldwide, affecting nearly half of the adults. Periodontitis is a major health problem reducing the quality of life. Not only does it cause tooth loss, disability, masticatory dysfunction, poor nutritional status, and compromised speech, but it is also independently associated with systemic chronic inflammatory diseases including atherogenic cardiovascular disease, type 2 diabetes mellitus, rheumatoid arthritis, chronic kidney disease, obesity, and chronic obstructive pulmonary disease [1,2,3].

Periodontitis is predominantly a bacterial infection involving the accumulation of different bacteria on the non-shedding surfaces. In susceptible patients, dental plaque comprises periodontal pathogenic microorganisms which initiate and trigger a dysfunctional inflammatory immune response, which results in the destruction of the underlying supporting tissues. The host response to the initiating periodontopathogens is mostly a genetically determined non-modifiable risk factor. However, modifiable or eliminative risk factors play an important role in changing the susceptibility or resistance of individuals to the disease. Such risk factors are smoking, poorly controlled diabetes mellitus, obesity, stress, osteopenia, and inadequate intake of calcium and vitamin D. [1]. The most important modifiable local risk factor is poor oral hygiene, which may initiate the infective inflammatory process in periodontal tissues.

Treatment of periodontitis involves a fine balance of various non-surgical and surgical methods carried out in order to reduce periodontal pocket depth, access residual plaque, initiate the regeneration of periodontal supporting tissues and decrease the risk of disease progression. Subgingival anti-infective therapy performed together with self-performed plaque control provides significant benefits in clinical parameters and improvement of systemic inflammatory markers [4].

Non-surgical periodontal treatment consists of professional removal of plaque and calculus, elimination of plaque retentive factors, oral hygiene instruction, chemical plaque control and antibiotic medication. Treatment of teeth with tooth/site-dependent factors (e.g., the presence of plaque, anatomic features, deep pocket, furcation involvement, intrabony defect) is less predictable and bears an elevated risk of the progression of the breakdown of periodontal tissues [5]. Mechanical therapy alone may have limited effect on some periodontopathogens and fail to eliminate them in ecological niches (e.g., infected epithelial and connective tissue pocket wall, scratches left after mechanical debridement, resorption lacunae, accessory root canal, dentinal tubules, dorsal part of the tongue, tonsils, oral mucosa, etc.). These limitations indicate the importance of the employment of pharmacologic agents in periodontal therapy [4].

Pharmacologic therapies are based on the administration of antimicrobials (including antiseptics and local delivery or systemic regimen of antibiotics) and probiotics and host modulation. Systemic antimicrobials should be applied adjunctively to mechanical non-surgical treatment—preferably in patients with aggressive or recurrent forms of periodontitis—and may provide additional benefits in case of very deep pockets or specific microbial infections. Systemic antimicrobial therapy is the most effective when the mechanical disruption of the subgingival biofilm is performed during subgingival debridement. The combination of amoxicillin and metronidazole or ciprofloxacin and metronidazole show greater clinical effectiveness than monotherapy [4,6].

The local delivery of antimicrobial drugs may reduce the systemic adverse effects and provide effective drug concentrations in the periodontal pockets to eliminate the pathogens from the subgingival area [7,8]. Local application of antibiotics has been advocated for patients with localized lesions or a limited amount of non-responding or recurrent sites [4]. In the literature, many articles are about the development of various types of delivery systems (fibers, films, gels, strips, injectable systems, microparticles) containing different active agents (tetracycline, doxycycline, chlorhexidine, clindamycin, metronidazole, amoxicillin, povidone-iodine) [7,9,10,11,12,13,14,15,16,17,18,19,20,21,22].

In the last 10–15 years, lipid drug delivery received considerable attention due to advancement in the field. Administration of lipid materials is most prevalent in solubility enhancing, lymphatic transport targeting, and/or intestinal transport modulation aiming the increased bioavailability of drug formulations, but creating sustained release systems, covering the bitter or bad taste of active ingredients or protecting drug molecules susceptible to different environmental factors may also be a purpose for the application of lipid excipients [23].

In periodontal therapy, using lipid formulations may provide the prolonged liberation of hydrophilic antimicrobial agents, while protecting them from decomposition in hydrophilic media and masking their unpleasant taste.

The aim of the recent study was to develop a lipid-based formulation, which can be inserted into the periodontal pocket, has mucoadhesive features and provides sustained delivery of antimicrobial agents, such as amoxicillin, metronidazole and zinc derivatives (zinc hyaluronate, and zinc gluconate). Due to the fact that the applications site possesses special qualities, the following requirements were considered to be met by our drug delivery system: to have a softening point at body temperature to help the adaptation of the dosage form to the shape of periodontal pockets; to be biodegradable to let the delivery system vanish slowly from periodontal pockets as healing commences; to be biocompatible not to trigger further inflammatory processes; and to provide sustained drug release for at least one week. In addition to softening, the purpose of the lipid composition was to create an anhydrous medium, which could protect the antimicrobial agents (amoxicillin) from decomposition and can have a taste-masking effect in the case of metronidazole. This paper also aims to investigate the properties of these delivery systems. During the development, different investigational methods were employed in order to examine the properties of the delivery systems. The measurements carried out included differential scanning calorimetry (DSC), X-ray diffraction (XRD), and mucoadhesion measurements, and also drug diffusion studies and antimicrobial effectiveness investigations.

## 2. Materials and Methods

### 2.1. Materials

Suppocire BP (SBP) pellets (Gattefossé Ltd., Saint-Priest, France), Methocel E4M HPMC (Colorcon Ltd., Dartford, United Kingdom), Kolliphor RH40 (KP) (BASF ChemTrade GmbH, Ludwigshafen, Germany), amoxicillin (AMX) (Antibióticos de León S.L.U., León, Spain), cetostearyl alcohol (CA), metronidazole (MZ), zinc gluconate (ZnGlu) (Ph. Eur. 8., Hungaropharma Plc., Budapest, Hungary), and zinc hyaluronate (ZnHA) (Richter Gedeon Ltd., Budapest, Hungary) were used to create the delivery systems.

For the mucoadhesion and drug dissolution tests, a phosphate-buffered saline (PBS) solution was prepared by dissolving 8 g/dm^3^ NaCl, 0.2 g/dm^3^ KCl, 1.44 g/dm^−3^ Na_2_HPO_4_ · 2 H_2_O and 0.12 g/dm^3^ KH_2_PO_4_ (Ph. Eur. 8., Hungaropharma Plc., Budapest, Hungary) in distilled water. The pH was adjusted to 7.4 by adding an adequate amount of 0.1 M HCl. The mucin (porcine gastric mucin type II, Sigma-Aldrich, Saint Louis, MO, USA) was used in the form of an 8 *w*/*w%* suspension.

### 2.2. Composition of the Drug Delivery Systems

The exact composition of different formulations can be found in Table 1. Five main components were used to create the delivery systems. A lipophilic component SBP was chosen as lipid base and CA was used as a structure-building component. All of the lipid components can be safely used on mucosae, where the SBP can soften at body temperature providing the accommodation of the dosage form to the periodontal pocket.

Cetostearyl alcohol (CA) consists of mainly stearyl and cetyl alcohols; it is a mixture of solid alcohols, it was used to increase the softening temperature of the formulations.

KP, a polyoxyethylene 40 castor oil derivative, was used as a surface-active agent, which helps the wetting of the formulation in the periodontal pocket; thus, it promotes its swelling and erosion.

HPMC (hydroxypropyl methylcellulose) and ZnHA were applied as gelling and mucoadhesive components. During the erosion of the formulations, they can help to prolong the retention time of the systems in the periodontal pockets. HPMC is often used as a mucoadhesive agent in oral formulations such as films [24] or buccal tablets [25] to increase the residence time of the delivery system, while ZnHA is regularly applied in topical gels to accelerate the healing of wounds of different etiologies and to decrease the chance of a possible bacterial superinfection [26], or may be used as a mucoadhesive agent in ophthalmic solutions to lengthen clearance and provide higher efficiency [27].

AMX or MZ, which are regularly applied orally for PD treatment, were incorporated into the vehicles. ZnGlu was also incorporated into one of the formulations as an antimicrobial agent. Zn salts have a known antibacterial effect, which can be useful in periodontitis treatment.

All the formulations were created by a melting and homogenation method. At first, the two lipophilic components (cetostearyl alcohol and Suppocire BP) and the surfactant (Kolliphor RH40) were melted together at 70 °C on a hot plate (IKA-WERKE RCT B, ETS-D4, IKA Werke GmbH and Co. KG, Staufen im Breisgau, Germany), then the polymer (zinc hyaluronate or Methocel E4M) was suspended and homogenized with an overhead stirrer at 50 RPM (Digital Overhead Stirrer–DLH, VELP Scientifica, Usmate Velate, Italy). The antimicrobial agent(s) (amoxicillin, metronidazole, zinc gluconate) was/were dispersed and homogenized at 50 °C to avoid thermal decomposition [28]. Delivery systems were created by molding the melted formulations into round shaped silicone molds. Cylindrical drug delivery systems were 1.5 mm in thickness and 9 mm in diameter.

### 2.3. Differential Scanning Calorimetry (DSC) Measurements

The softening temperature and the melting point of the formulations were determined by differential scanning calorimetry measurements with a Mettler-Toledo DSC 821e instrument in an argon atmosphere (100 mL/min). The temperature was raised from +5 °C to +100 °C by 5 °C per minute. Ten milligrams of the samples were put in 40 µL aluminum pans. The tops were holed, then the pans were sealed.

### 2.4. X-ray Powder Diffraction (XRD) Analysis

Diffractograms of the raw materials (SBP, CA, KP, MZ, AMX, HPMC, ZnHA, ZnGlu) and the formulations were obtained with a Bruker D8 Advance diffractometer (Bruker AXS GmbH, Billerica, MA, USA) system with Cu K λI radiation (λ =1.5406 Å). Each sample was scanned at 40 kV and 40 mA in the interval of 3°–40° 2θ, at a scanning speed of 0.1/s and a step size of 0.010°.

### 2.5. Mucoadhesion Measurements

Adhesion tests were performed with a TA-XT Plus (Texture analyzer, ENCO, Spinea, Italy) instrument equipped with a 1 kg load cell and a cylinder probe with a diameter of 1.0 cm. Twenty milligrams of the sample attached to the cylinder probe was placed in contact with a filter paper disc impregnated with 50 µL mucin dispersion (8 *w*/*w%*, prepared in PBS (pH = 7.4)). A 2500 mN preload was applied for 3 min (the compression speed was 2.5 mm min^–1^). The cylinder probe was then moved upwards to separate the sample from the substrate at a prefixed speed of 2.5 mm min^–1^. Polymer solutions, prepared by dissolving the solid polymers in PBS, were analyzed during the mucoadhesion measurements where ZnHA and HPMC concentrations were 0.5, 1, 2, 5, and 10 *w*/*w%*. Ten parallel measurements were performed.

### 2.6. Investigation of the Swelling and Erosion of Formulations

A gravimetrical analysis was carried out in order to investigate the water absorption capacity of the different formulations, and to examine the effects of the mucoadhesive polymers on the erosion profile of the delivery systems. Prior to the measurements, the formulations were weighed with an analytical scale and put in 10 mL of PBS solution thermostated at 37 °C for various time periods (0.5, 1, 2, 4, 6, 8, 24, 36, 48, 72, 96, and 168 h). Subsequent to the designated time period, the formulations were weighed again, which was followed by drying at room temperature. After the formulations had totally dried out, a third weight measurement was carried out. Evaluation of the changes in the mass of the formulations could help the determination of the absorption capacity and the swelling related erosion profiles. For each sampling time three parallels were applied. 

### 2.7. Drug Dissolution Tests

The in vitro drug release profiles of formulation nos. 1, 2, 3, and 6 were determined. The compositions were weighed with an analytical scale, put in 50-mm-long dialysis tubes (Spectra/Por^®^ Standard RC tubing, MWCO: 12-14 kD) and sealed with closures. The tubes containing the formulations were placed in 7.5 mL of PBS solution thermostated at 37 °C. Drug release was investigated for seven days and three parallel measurements were carried out. One milliliter of samples was taken (at 0.5, 1, 2, 4, 6, 10, 24, 30, 48, 72, 96 and 168 h) and were replaced with 1 mL of PBS solution. The active agents AMX and MZ were separated with an HPLC system (where necessary) and quantified by a UV spectrophotometer attached to the HPLC at 230 nm.

The solution obtained was filtered through a 0.20 µm polyethersulfone syringe membrane filter and injected directly into the HPLC system. The AMX and/or MZ content was quantified with a Merck-Hitachi LaChrome Elite HPLC (Hitachi High Technologies America, Inc., Schaumburg, IL, USA). AMX and MZ were measured on a Kinetex 250 mm × 4.6 mm column packed with 5 µm EVO C18, 100 Å (Phenomenex Inc., Torrance, CA, USA). Elution was performed with 20:80 (*v*/*v*) Methanol-NaH2PO4 (0.05 M) at a flow rate of 1 mL/min. Prior to the elution, the eluent was degassed and filtered through a 0.45 µm pore-sized glass filter funnel. The run time was seven minutes. Detection was performed via absorption at 230 ± 4 nm. 3 µL of sample were injected, and the elution was carried out at a sample temperature of 25 °C and at a column temperature of 25 °C.

Quantitative determination was achieved by comparison with the spectra of standards. The stock solutions of AMX and MZ (2 mg/mL) were prepared in PBS and stored at 4 °C. The stock solutions were used within 24 h. Working standards (0.5, 1, 1.5, and 2 mg/mL) were prepared freshly by diluting the stock solution with the mobile phase prior to the HPLC analysis. Calibration plots were freshly prepared and were highly linear (*R*^2^ (AMX) = 0.9990 and *R*^2^ (MZ) = 0.9998). Limit of detection (LOD) and limit of quantification (LOQ) of the quantitative determination was 3.308 and 10.020 µg/mL, respectively, while in the case of amoxicillin and metronidazol, the resolution (R_S_) was 3.10.

Drug recovery tests were also performed, where the incorporated metronidazole was extracted with water at the melting point of the lipid formulations. The results of the extracted drug amounts corresponded to the incorporated drug amounts (99.50 ± 0.30%).

### 2.8. Microbiological Investigation

Microbiological study was conducted in order to measure the antibacterial effectiveness of the developed formulations. Typical periodontopathogenic bacteria: *Fusobacterium nucleatum* (ATCC^®^ 25586™), *Parvimonas micra* (ATCC^®^ 33270™), *Eikenella corrodens* (ATCC^®^ 23834™), *Porphyromonas gingivalis* (ATCC^®^ 33277™), *Aggregatibacter actinomycetemcomitans* (ATCC^®^ 29524™), and *Prevotella intermedia* (118710) control strains were used. A 1 McFarland standard concentration bacterial suspension of each bacterial strain was made separately with 0.9% NaCl solution (in suspension it is equivalent to approximately 3 × 108 colony forming units/mL). The suspension was spread onto a horse blood agar plate, where then formulations were placed on. The concentration of the active agent(s) was 15 *w*/*w%*: 15 *w*/*w%* of AMX or MZ alone (formulations 1 and 2), or 7.5 *w*/*w%* of both AMX and MZ (formulation 3). The delivery systems made with ZnHA contained 3 *w*/*w%* ZnHA (formulation 4) or 3 *w*/*w%* ZnHA and 0.2 *w*/*w%* ZnGlu (formulation 5) or 3 *w*/*w%* ZnHA and 15 *w*/*w%* MZ (formulation 6). After 24 h of incubation in anaerobic conditions, the diameter of the inhibition zones was measured. The formulations were then put on a new horse blood agar plate, also inoculated with 1 McFarland standard concentration freshly made bacterial suspension of each of the above-mentioned bacterial strains. The plates were then put in an anaerobic chamber for 24 h. This was repeated until no inhibition zone could be detected.

## 3. Results and Discussion

### 3.1. Differential Scanning Calorimetry (DSC) Measurements

DSC was used to determine the softening temperature and the melting point of the formulations.

**Figure 1:**
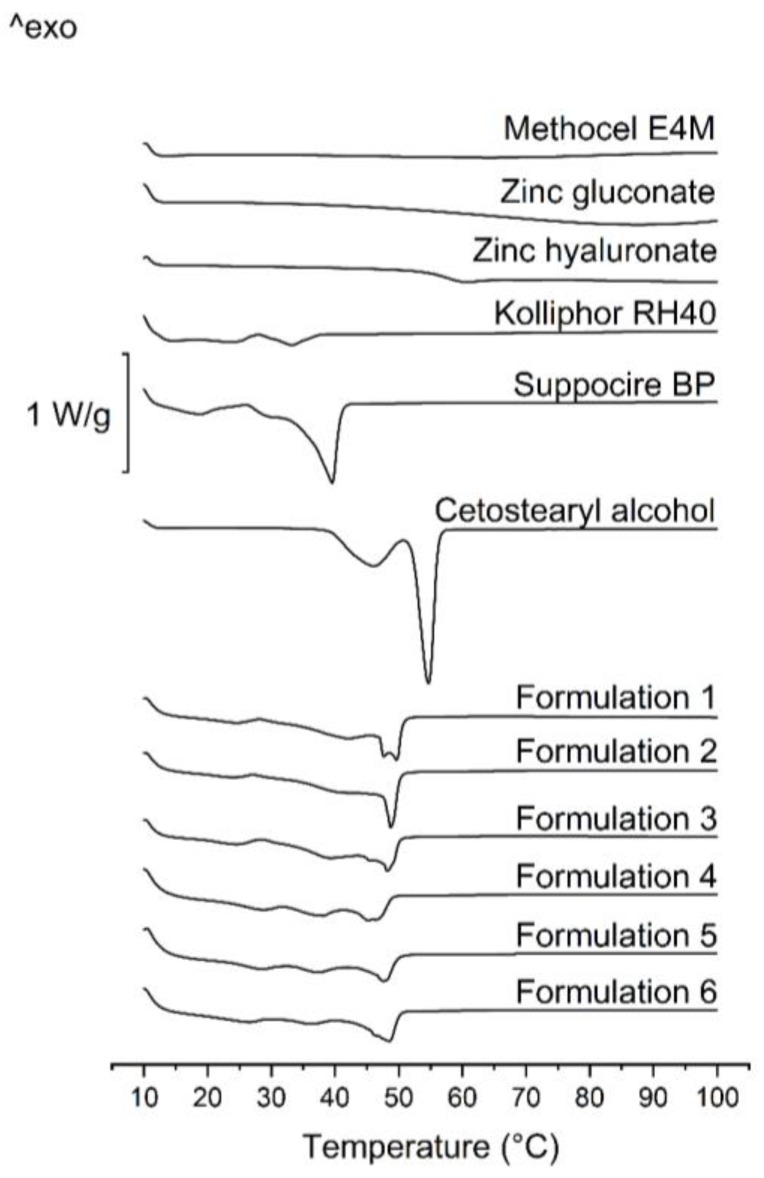
DSC curves of pure components (Kolliphor RH40, Suppocire BP and Cetostearyl alcohol) and formulations containing 15 *w*/*w%* of amoxicillin, 15 *w*/*w%* of metronidazole, 7.5 *w*/*w%* of amoxicillin and metronidazole, 3 *w*/*w%* of zinc hyaluronate, 3 *w*/*w%* of zinc hyaluronate and 0.2 *w*/*w%* of zinc gluconate, and 3 *w*/*w%* of zinc hyaluronate and 15 *w*/*w%* of metronidazole.

The lipid base of the compositions was SBP, which is often used as a base for suppositories [29]. In our measurements, it has a melting point at 39.08 °C (Figure 1).

Cetostearyl alcohol is mostly used as a surfactant, a co-surfactant or as a viscosity increasing agent in semisolid formulations. According to our DSC measurements, the melting point of the pure component is at 61 °C (Figure 1.). In our earlier study [30], we presented that where CA remains partially in a crystalline state; thus, sustained drug release could be achieved.

The results of DSC measurements show that Kolliphor RH40 has a congealing temperature approximately between 20—30 °C (Figure 1), which corresponds with the literature data [29].

No sharp peaks could be perceived on the curves belonging to the polymers (zinc hyaluronate and Methocel E4M), and zinc gluconate in this temperature range (10—100 °C). Glass transition of these components starts over 50 °C (Figure 1.)

Formulation nos. 1–6 were examined to investigate the possible modifying effect of the incorporated drugs and excipients on melting point. Antibiotics or other suspended materials may partially dissolve in the lipid base, therefore, the effect of various incorporated components of different amounts on the melting point was also investigated.

In all formulations, the sharp peaks of SBP and CA disappeared, even the two peaks of CA morphed into one, and shifted to temperatures between the melting points of the two pure components. At approximately 30 °C, a moderate melting can be observed, but total melting only occurs between 40 and 50 °C. A moderate melting starts at 30 °C, which supports the softening of the systems, but, as there are components with melting points at about 50 °C, which suggests the presence of a coherent structure at body temperature; thus, total melting of the formulations will not occur, and sustained drug release may be possible.

The type of the drug did not influence the melting point greatly, but differences in the shape of the curves are noteworthy. In the case of formulations containing zinc salts or amoxicillin, peaks at approximately 50 °C (probably the two fused peaks of CA) broadened, meaning an earlier onset of melting, while metronidazole containing ones show sharper peaks.

By the thorough analysis of the DSC results, we could conclude that no peaks of any curve belonging to different formulations could be associated with the decomposition of any component, thus, the constituents are expected to be stable in this temperature range.

### 3.2. XRD Analysis

Active ingredients were suspended in the melted lipids. XRD analyses were carried out to investigate their final state (suspended or dissolved). The presence of solid crystals is indicated by sharp peaks in the diffractograms, while dissolved components, as they do not possess a crystalline structure, are not detectable. The amorphous structure is often a substantial feature of polymers; thus, the crystalline structure and its characteristic peaks are absent from the diffractograms.

**Figure 2:**
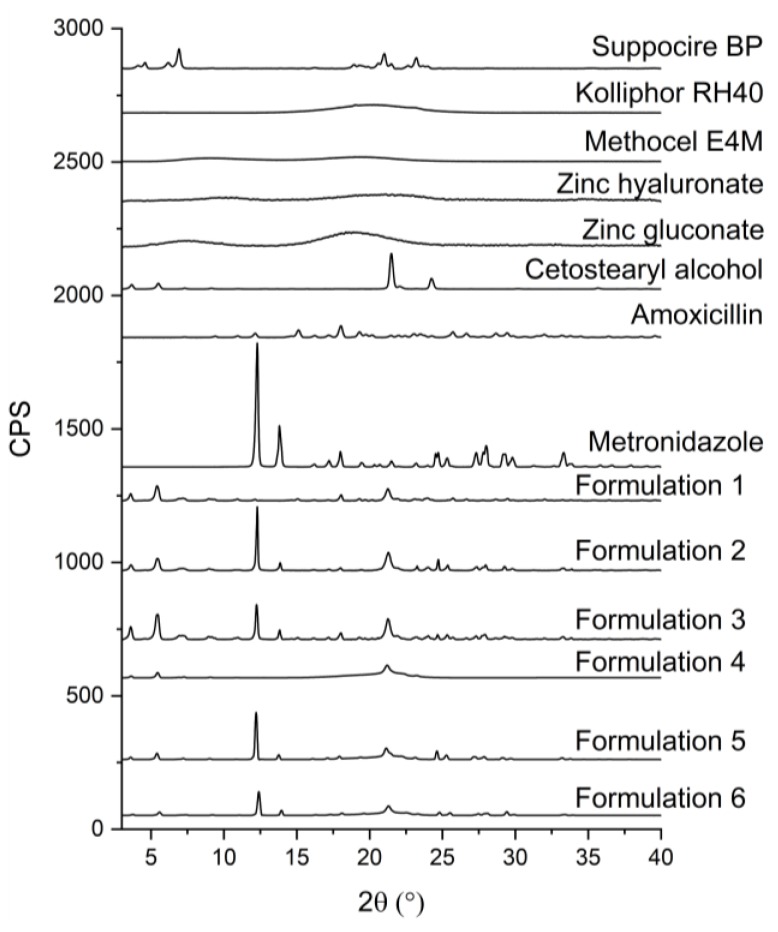
Diffractogram of pure components (Kolliphor RH40, Suppocire BP and cetostearyl alcohol, amoxicillin, metronidazole, zinc hyaluronate, zinc gluconate, Methocel E4M) and formulations containing 15 *w*/*w%* of amoxicillin, 15 *w*/*w%* of metronidazole, 7.5 *w*/*w%* of both of amoxicillin and metronidazole, 3 *w*/*w%* of zinc hyaluronate, 3 *w*/*w%* of zinc hyaluronate and 0.2 *w*/*w%* of zinc gluconate, and 3 *w*/*w%* of zinc hyaluronate and 15 *w*/*w%* of metronidazole.

All of the components, except for HPMC, Kolliphor RH40, zinc hyaluronate, and zinc gluconate, have a crystalline structure according to the XRD diffractograms presented in Figure 2.

SBP and CA have similar molecular structures, which will result in analogous diffractograms. This means that in the diffractograms of the formulations containing all of the components, it is hard to differentiate between the two lipid components. The more intensive peak of CA could overlap with and cover the peak of SBP at the same 2θ value in the preparations. This peak, which belongs to CA, has decreased in intensity in the diffractograms of the formulations, which implies that the crystallinity of CA has decreased, which is in accordance with the DSC results, where the two peaks of pure CA have fused together and shifted to a lower temperature range.

A characteristic peak linked to SBP at the 2θ value of approximately 5° is present in all formulations with varied intensity, also indicating the crystalline structure of the lipid base.

The characteristic peaks of AMX and MZ could be separated from those of the lipid components. The presence of characteristic peaks in the diffractograms is the sign of solid particles in the formulations. AMX and MZ have a characteristic peak at the 2θ value of 18.1° and 12.3°, respectively. Peaks at the mentioned 2θ values could be observed in the diffractograms of formulations containing AMX or MZ.

The presence of ZnHA or ZnGlu in the preparations could not be demonstrated by this experiment because of their amorphous structure to which no characteristic peak belongs in an XRD diffractogram.

The partially crystalline form of the lipid matrix (CA and SBP) could contribute to the coherent structure of the system, which does not melt at body temperature, thus providing sustained release of antibiotic compounds, while the suspended form of the drugs and antimicrobial agents may provide a more stable form, therefore protecting the drugs from the negative effects of the environment and decomposition as well.

### 3.3. Investigation of the Mucoadhesive Properties of the Applied Polymers

Subsequent to insertion into the periodontal pocket and wetting of the lipid system, the mucoadhesive polymers at the interface of the lipid and the periodontal fluid get hydrated, swell, and form a gel layer, providing adhesion to the application site.

The mucoadhesive properties of the polymers were investigated by means of a tensile test, which is an in vivo-like mucoadhesion method because it mimics the adhesion to a biological surface. Adhesive force and work were determined on the basis of the force-distance curve obtained during the tensile test (Table 2). In this measurement, a larger concentration range of polymers was investigated. The larger concentration range of polymer solutions could mimic the gel layer formed at the surface of the lipid formulation; therefore, the mucoadhesion properties of the delivery system.

In our measurements, in both cases, the adhesive force and work continuously increased with increasing polymer concentrations; ZnHA showed remarkable adhesion over the whole investigated polymer concentration range, indicating better mucoadhesion to the model surface; thus, ZnHA may provide a better mucoadhesive feature for the lipid formulations than HPMC, and the multipotent (adhesive and antimicrobial) characteristics of the former excipient can be beneficial in periodontal therapy.

### 3.4. Swelling and Erosion Profiles of Delivery Systems

In addition to melting and softening, swelling and erosion of the lipid formulation can also affect drug release. In this case, the swelling of the polymer can result in a gel layer on the surface of the delivery system, and then this layer can be slowly removed thanks to the elimination mechanisms in the oral cavity and periodontal pocket. In addition to this mechanism, the melted lipid and the surfactant can also be eliminated from the formulation by emulsification with the produced oral biological medium, the gingival crevicular fluid.

In our experiments the swelling ratio (%) was calculated as follows [31]:

Swelling ratio (%) = *m*_s_ / *m*_o_ × 100% (1)

where *m*_s_ is the mass of the formulations after immersed in water for the specified time and *m*_o_ is the mass of the original formulations (prior to the immersion into water).

**Figure 3:**
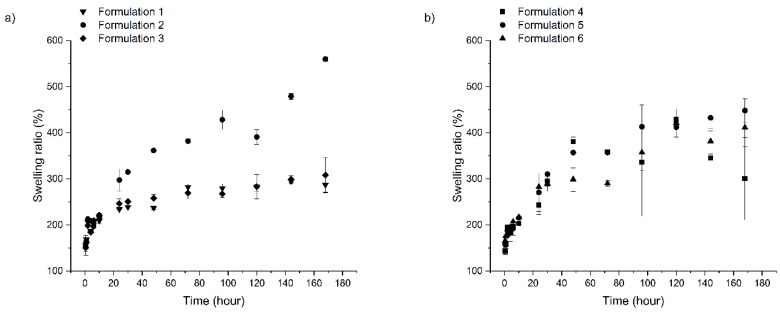
Swelling profiles of formulations: (**a**) 1–3 and (**b**) 4–6.

**Figure 4:**
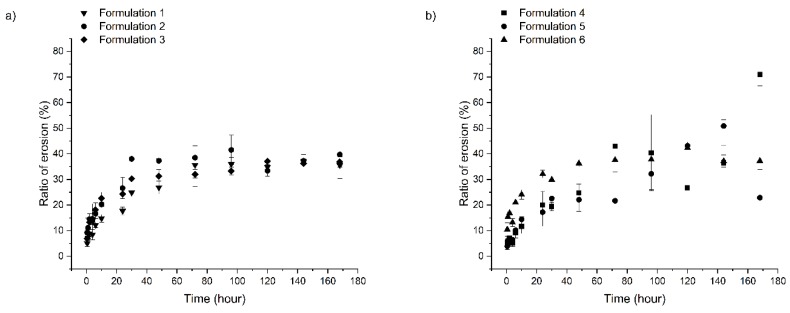
Erosion of formulations: (**a**) 1–3 and (**b**) 4–6.

During the measurements, delivery systems were immersed into purified water to investigate the swelling and erosion properties of the different preparations, because different antimicrobial agents and mucoadhesive polymers are incorporated into the systems. The swelling ratio of the different compositions can be observed in Figure 3a,b, while the erosion profiles are shown in Figure 4a,b.

Based on the swelling profile, two groups can be distinguished. Swelling ratio of formulations containing ZnHA and/or a higher amount of MZ shows a higher water absorption capacity than formulations with AMX or lower concentrations of MZ and HPMC as polymer; the former group had a maximum swelling ratio of approximately 560%, while the latter group was only approximately 300%.

This phenomenon may be driven by the different swelling and solubility of components: i) ZnHA is a material possessing a strong water absorption capacity, and ii) MZ owns a sparingly water-soluble feature but better than amoxicillin. Because of the higher water absorption-capacity of ZnHA and better solubility of MZ, water could access the lipid structure with ease resulting in higher swelling of formulations; ZnHA may bind a very large amount of water and break the coherent lipid structure, and through the generated superficial microgaps more ZnHA particles become accessible to water, thus binding more medium. MZ may work in a highly similar way, but in this case the applied polymer (HPMC) binds the water. The better solubility of MZ compared to AMX, also contributes to quicker dissolution from the systems, and the dissolved MZ (leaving capillaries in the structure) may provide water with a trouble-free entry accessing polymer particles incorporated into the delivery systems. The more drug is dissolved from the preparations, the more capillaries are made, and through more capillaries, more polymer particles may be accessed, which results in a higher water binding. When ZnHA and a high concentration of MZ (15 *w*/*w%*) are applied together, no extra swelling can be observed. This phenomenon can be explained by the structure of the formulations, the swelling power of the polymers may not be vigorous enough to destroy the coherent solid structure of the delivery system; thus, combining the two components (MZ and ZnHA) did not result in over-swelling.

AMX and lower concentrations of MZ are not capable of generating the same effect due to the creation of fewer capillaries because of the poorer solubility of amoxicillin compared to MZ and the lower concentration of MZ; therefore, the swelling of those systems remains below the level provided by the formulations having ZnHA or more MZ incorporated into them.

Accordingly, the swelling profiles of the different formulations may be dependent on the incorporated materials, their attributes and the strength of the coherent lipid structure.

Erosion ratio (%) was calculated from the following equation [30]:

Erosion ratio (%) = *m*_o_−*m*_d_/*m*_o_ × 100% (2)

where *m*_o_ is the mass of the original formulations and m_d_ is the mass of the dried formulation after the swelling and erosion measurements.

In contrast with the results of the swelling measurements, the erosion of different compositions are not highly different from each other (Figure 4a,b). The weight of all formulations has decreased to approximately 60% of that of the original ones. Two main mechanisms may control the erosion of the drug delivery systems. One is the emulsification of components melted at body temperature (37 °C) with water. The other is the swelling power of incorporated polymers, destroying the coherent structure, and the erosion of the gel layer formed at the surface. The similarities in the erosion profiles can highlight that the destruction of delivery systems may be more likely to be defined by the emulsification of the melted lipid components, as the lipid composition and ratio and, therefore, the basic structure of the different compositions, are almost the same. The swelling experiments also suggest the same findings, because the strength of the coherent structure could limit the swelling of polymers.

### 3.5. Investigation of Drug Release from Formulations

Drug dissolution measurements were carried out in order to evaluate the drug release profiles of the prepared formulations which contain MZ. An HPLC method was used to quantify the dissolved drug from the preparations.

**Figure 5:**
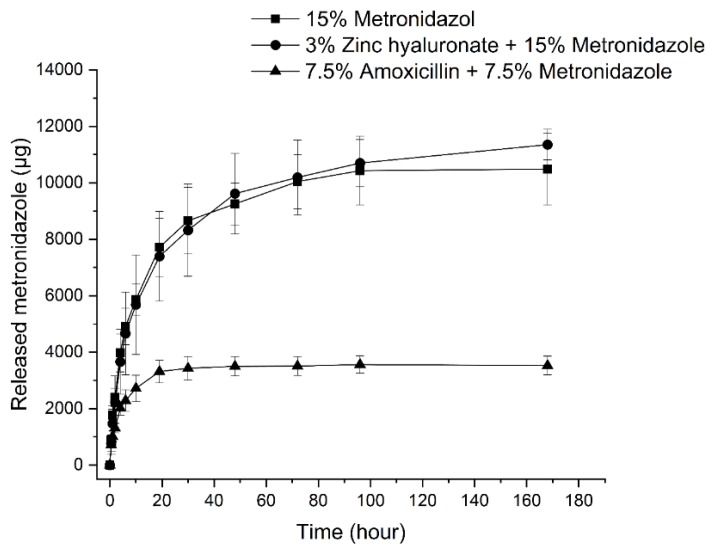
Released amount of metronidazole (μg) from formulations with different compositions (15 *w*/*w%* metronidazole, 3 *w*/*w%* zinc hyaluronate and 15 *w*/*w%* metronidazole, and 7.5 *w*/*w%* amoxicillin and 7.5 *w*/*w%* metronidazole).

The amount of released MZ from formulations containing the antibiotic drug is shown in Figure 5. Preparations containing 15 *w*/*w%* MZ and HPMC or ZnHA have shown similar drug release profiles. A plateau phase commencing at approximately at the 100th hour is indicated in both cases, which is in accordance with a total drug release.

However, the drug release curves of formulations containing 7.5 *w*/*w%* MZ indicates that only one-third of the amount was liberated from the preparations despite the fact that half of the amount had been incorporated into the compositions.

The drug release mechanism can be characterized with the following exponential equation:

*M*_t_/*M*_∞_ = *k* × *t^n^* (3)

where *M*_t_/*M*_∞_ is the fraction of drug released, *k* is the kinetic constant and *n* is the release exponent describing the mechanism of the release.

On the basis of literature data, in case of swelling controlled cylindric drug delivery systems, if the release exponent is 0.45, the dug release mechanism is a Fickian diffusion, while the release exponent is between 0.45 and 0.89, the release corresponds to anomalous (non-Fickian) diffusion [32]. In our case, the highly swellable formulations (Formulations 2 and 6) showed non-Fickian diffusion, which indicates that Fickian diffusion coupled with the swelling of the incorporated polymer. In the formulation where swelling is moderated (Formulation 3) alone Fickian diffusion characterizes the drug release (*n* = 0.4567) (Table 3).

The phenomenon detailed above in Section 3.4. may be in the background of such results. MZ, which is a more soluble material in water compared to AMX, could be liberated more quickly from formulations creating capillaries, which contribute to a higher swelling and therefore allow water to access all the suspended drug in the delivery systems. On the contrary, when smaller amounts of MZ are incorporated into the delivery systems with AMX—a substance with lower water solubility—fewer capillaries may be formed during drug release, permitting less water to penetrate the systems and resulting in non-complete drug dissolution.

The application of ZnHA did not change the release profile, which can be explained by the similar swelling and erosion profiles of the formulation containing 15 *w*/*w%* MZ with and without ZnHA. 

Summarizing the results of drug dissolution testing, a sustained release of drugs could be achieved with these compositions, and the main factors affecting drug release are swelling (driven by the applied hydrophilic components such as polymer, active ingredients and their concentration) and the strength of the coherent lipid structure. 

### 3.6. Microbiological Investigation

Numerous different species of microorganisms may be present in the oral cavity or on the dental surfaces. The composition of the oral microbiota varies widely from person to person, from place to place and naturally with dietary habits [33,34].

Many journal articles aim to define the species of bacteria which have a contribution to the initiation and progression of periodontal disease [33,34,35,36,37,38,39,40,41], but the high number of different strains and cultivation difficulties make it almost impossible. Moreover, only a small percentage of the subgingival oral microbiota has a role in disease pathogenesis [42,43]. Pathogen organisms which could be linked to and may have correspondence to the disease are the following: *Porphyromonas gingivalis*, *Prevotella intermedia*, *Bacteroides forsythus*, *Actinobacillus actinomycetemcomitans*, *Treponema denticola*, *Tannerella forsythia*, *Fusobacterium nucleatum*, *Fusobacterium periodonticum*, *Prevotella intermedia*, *Prevotella nigrescens*, *Parvimonas micra*, *Campylobacter gracilis*, *Campylobacter rectus*, *Campylobacter showae*, *Eubacterium nodatum*, *Streptococcus constellatus*, *Eikenella corrodens*, *Streptococcus* spp., etc. [33,42,43,44,45]. According to Kolenbrander et. al. [45], these species can be separated into two categories concerning plaque formation. Species in the first category—initial colonizers—are thought to stick to the tooth surface and proliferate. The second group—late colonizers—binds to the first group of bacteria via different interactions.

In our formulations, AMX or MZ was incorporated in 15 *w*/*w%* concentration, and a system containing 7.5/7.5 *w*/*w%* AMX and MZ was also compiled. In addition to these formulations, ZnHA, ZnHA and ZnGlu, and ZnHA and MZ containing ones were also created and investigated.

AMX, which is a semi-synthetic penicillin derivative, is known to be effective against many aerobic or anaerobic Gram-positive and Gram-negative bacteria [13,46].

MZ is an antibacterial agent, included in the nitroimidazole group. Numerous bacteria and protozoa—even anaerobes—are known to be susceptible to MZ [7,47].

In periodontal disease therapy, MZ and AMX may be used as an adjunct to scaling and root planning therapies locally or systematically [48,49,50].

Hyaluronic acid is a naturally occurring polysaccharide of the extracellular matrix of connective tissue, synovial fluid, and soft periodontal tissues as well. Its application in the treatment of the inflammatory process is established in different medical areas such as orthopedics, dermatology, and ophthalmology. In the treatment of periodontal diseases, hyaluronic acid shows anti-inflammatory and anti-bacterial effects [51]. 

Zinc salts may have a beneficial effect when used in mouth rinses, can possibly decrease plaque formation by inhibiting glycolytic enzymes and may prevent the attachment of bacteria to the tooth surface [52]. These salts administered in combination with other antimicrobial agents may show a synergism by means of antibacterial effect [53].

In view of the beneficial properties of hyaluronates, zinc hyaluronan was chosen as a combined mucoadhesive and antimicrobial agent for our other set of formulations. 

The antibacterial effectiveness of samples with Zinc hyaluronate was also measured to determine if the polymer has an antibiotic effect alone or in combination with other molecules like zinc gluconate or metronidazole.

In our microbiological investigation, where the antimicrobial activity of our formulations was measured, we used six different strains of oral pathogen bacteria, which may contribute to the initiation of periodontitis. These were the following: *Eikenella corrodens*, *Prevotella intermedia*, *Parvimonas micra*, *Fusobacterium nucleatum*, *Aggregatibacter actinomycetemcomitans*, and *Porphyromonas gingivalis*.

*Eikenella corrodens*—a facultative anaerobic, Gram-negative bacterium—is a human (mostly oral) pathogen. *E. corrodens* appears to be susceptible to beta-lactam antibiotics, but resistant to metronidazole [54].

*Parvimonas micra* (also known as *Peptostreptococcus micros* or *Micromonas micros*)—an anaerobic, Gram-positive coccus—is the member of the normal human gastrointestinal flora [55]. According to Rams et al., it shows high susceptibility to penicillin but metronidazole seems to be less effective against the bacterium [56].

*Prevotella intermedia*—a Gram-negative anaerobic bacterium with black pigments—is often connected with oral and subgingival diseases. P. intermedia shows susceptibility to penicillin and metronidazole [57].

*Fusobacterium nucleatum*—a mostly oral and periodontal anaerobic pathogen—can be linked to a variety of human diseases. Strains isolated from endodontic infections seem to be highly sensitive to amoxicillin and a little less susceptible to metronidazole [58,59].

*Aggregatibacter actinomycetemcomitans*—a Gram-negative, facultatively anaerobic rod—is frequently associated with most forms of periodontitis and numerous oral infections. Amoxicillin is effective against the bacterium, while it shows much less susceptibility to metronidazole [60,61].

*Porphyromonas gingivalis*—an obligately anaerobic, Gram-negative black-pigmented rod—is the most commonly linked microorganism to periodontal disease. According to susceptibility tests, *P. gingivalis* is sensitive to amoxicillin and metronidazole [62,63].

Agar plates were 90.0 mm in diameter, thus, the largest possibly detectable inhibition zone could be around 90.0 mm.

**Figure 6:**
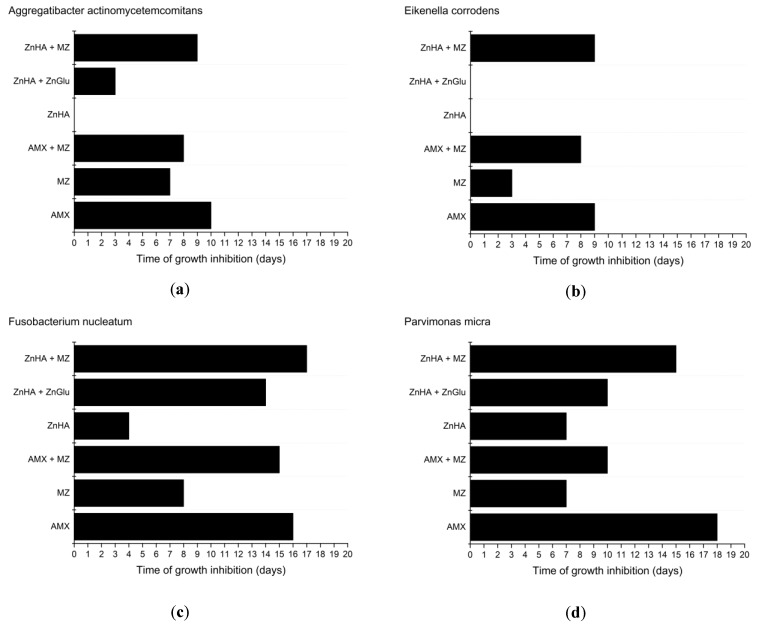

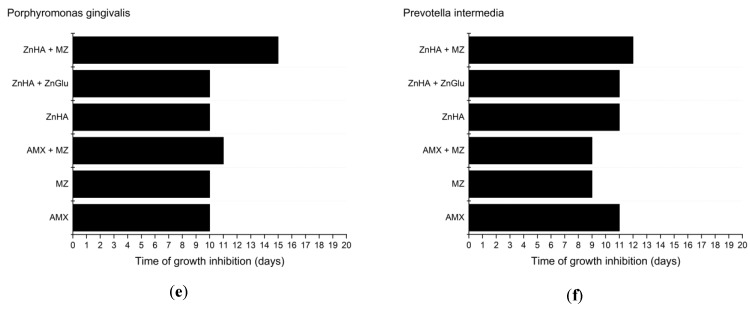
Effectiveness of delivery systems containing different antimicrobial agents against various oral anaerobic pathogenic bacteria: (**a**) *Aggregatibacter actinomycetemcomitans*; (**b**) *Eikenella corrodens*; (**c**) *Fusobacterium nucleatum*; (**d**) *Parvimonas micra*; (**e**) *Porphyromonas gingivalis*; and (**f**) *Prevotella intermedia*.

The results of the microbiological investigations can be found in Figure 6.

The largest measurable inhibition zone was 74.0 mm in diameter and was because of an amoxicillin containing formulation on an agar plate inoculated with *Eikenella corrodens* after the first 24 h.

According to our measurements, *Parvimonas micra* was the most sensitive microorganism to AMX containing formulations as the compositions could provide 17 days of effective drug release, while the least susceptible pathogen was *Eikenella corrodens* with only eight days of growth inhibition.

MZ susceptibility was slightly lower, as the effect against the most sensitive bacterium (*Porphyromonas gingivalis*) was only nine days. *Eikenella corrodens* was the least susceptible to MZ as on the first 2 days no growth could be detected, but after two days bacteria were growing all over the agar plate, and there was no inhibition zone around the formulation. This result is in accordance with the literature data, where it was established that *Eikenella corrodens* is resistant to MZ [46].

Compositions containing 7.5 *w*/*w%* of AMX and MZ did not provide larger inhibition zones than formulations containing only one antimicrobial agent as the antimicrobial effect lasted for shorter time periods. *P. gingivalis* was an exception: this bacterial strain was the only one with higher susceptibility to the combination of the two antimicrobial drugs. In most cases, the growth inhibition effect of combinations lasted longer than that of metronidazole. This could have been possible due to the more potent antimicrobial effect of AMX. Lower susceptibility to the combination of AMX and MZ compared to only AMX containing formulations may be due to the decreased concentration of AMX in the combination containing compositions and the lower susceptibility of bacteria to MZ.

Whereas MZ is less potent against *Aggregatibacter actinomycetemcomitans*, *Fusobacterium nucleatum*, and *Parvimonas micra* [56,58,59,60,61], with our formulations the growth inhibition effect can be provided for up to 3 days.

Susceptibility to ZnHA is different among various bacterial strains. *Aggregatibacter actinomycetemcomitans* and *Eikenella corrodens* show resistance to ZnHA, but combined with ZnGlu, a longer effect can be observed in case of *Aggregatibacter actinomycetemcomitans*. *Eikenella corrodens* remains unsusceptible to the combination of ZnHA and ZnGlu.

According to the results, the same susceptibility characterizes *Porphyromonas gingivalis* and *Prevotella intermedia* when using ZnHA alone or in combination with ZnGlu.

In case of *Parvimonas micra* and *Fusobacterium nucleatum*, the results show that higher efficiency may be achieved by administering a combination of ZnHA and ZnGlu instead of using only ZnHA.

The results imply that the combination of MZ and ZnHA provides a synergistic effect: formulations containing both ZnHA and MZ provided longer growth inhibition against the microorganisms than the active agents alone. The synergistic effect is most conspicuous against *Eikenella corrodens* where formulations with MZ only lasted for two days, and ZnHA had no antibacterial effect against the bacteria, while the combination showed a nine-day effect.

All in all, the outcome of the antimicrobial investigation of ZnHA supports that alone it may not be effective enough, but a combination with an antimicrobial drug (e.g., MZ) may provide a synergistic and—in this case—a longer positive effect.

While the in vitro dissolution of AMX could not be detected, because the antibiotic had decomposed thanks to the test environment (aqueous medium at 37 °C), the results of the microbiological investigation imply that AMX stays stable when incorporated into the lipid preparations, as it may provide 18 days of growth inhibition. (In this experiment, anaerobic environment was provided, and the agar plates were changed day-by-day, which enabled amoxicillin to remain stable in the aqueous agar medium.) This finding and the swelling experiments suggest that the combination of AMX and a highly hydrophilic polymer with strong water binding capacity may not be beneficial, because the sustained release of AMX could easily result in the decomposition of the drug, even in the formulation prior to its dissolution.

## 4. Conclusions

An anhydrous lipid-based drug delivery system for the treatment of periodontitis was successfully prepared and investigated. Possible administration and positive effect during disease treatment were confirmed by the results of different measurements.

According to the DSC measurements, the melting of formulations starts at body temperature, but total melting only occurs over 40 °C, which allows the presence of a coherent structure and provides sustained drug release.

X-ray diffraction analysis indicates that the antibiotics are in a suspended form in the compositions, and lipid components have a crystalline structure, which may also contribute to a prolonged drug release and antimicrobial effect.

Thanks to the complex composition, our lipid formulations are swellable and degradable systems. On the basis of our results, their swelling can be driven by the applied hydrophilic components (polymer, active ingredients), their concentration and the strength of the coherent lipid structure, while their erosion may be controlled mainly by the emulsification of the melted lipid components.

In formulations, where swelling is remarkable, Fickian diffusion is coupled with the swelling of the incorporated polymer. Because of this phenomenon, which is indicated in the shape of the diffusion curves, an anomalous (non-Fickian) diffusion model could be fitted to the diffusion curves. The results of drug release and antimicrobial investigations show that with AMX incorporated into the delivery systems, long drug diffusion can be obtained with a high antimicrobial effect. AMX can be protected against degradation in hydrophilic circumstances at the periodontal pocket by using these lipid formulations, which is suggested by our antimicrobial investigations. The sustained drug release of MZ can also be provided, which may enable taste-masking and direct application of MZ in the oral cavity without decreasing patient compliance. The combination of ZnHA and MZ improved the antimicrobial characteristics of formulations compared to formulations containing only ZnHA or MZ. These findings suggest that—when administered together—the two compounds have a synergistic effect and might be a good alternative for patients allergic to penicillin and their derivatives.

In order to improve the residence time of melted lipid formulations in the periodontal pocket, mucoadhesive polymers were applied. In our experiments, ZnHA had better adhesivity compared to HPMC, and, additionally, it has a potential antibiotic feature. Nevertheless, the application of a highly hydrophilic polymer with a strong water absorption capacity is questionable when the drug, e.g., AMX, is not stable in aqueous circumstances.

The developed and evaluated lipid formulations containing antimicrobials may offer a solution to such problems of local periodontal therapy as long-lasting effect. Moreover, the combination of MZ and ZnHA can provide an effective antimicrobial therapy for penicillin-allergic patients.

## Figures and Tables

**Table 1:** Composition of formulations 1–6. All values are given in *w*/*w%*.

Formulation No. Component	1	2	3	4	5	6
Suppocire BP	33%	33%	33%	47%	46.8%	32%
Cetostearyl alcohol	40%	40%	40%	40%	40%	40%
Kolliphor RH40	10%	10%	10%	10%	10%	10%
Methocel E4M	2%	2%	2%	-	-	-
Metronidazole	-	15%	7.5%	-	-	15%
Zinc hyaluronate	-	-	-	3%	3%	3%
Amoxicillin	15%	-	7.5%	-	-	-
Zinc gluconate	-	-	-	-	0.2%	-

**Table 2:** Adhesive force and work of the gels at different polymer concentrations. Mean values and SD, *n* = 10. (ZnHA = Zinc hyaluronate; HPMC = Methocel E4M, hydroxypropyl methylcellulose).

Polymer	Polymer Conc. (%)	Adhesive Force (mN)		Adhesive Work (mN.mm)	
		Mean ± SD		Mean ± SD	
ZnHA	0.5	1579.0	71.1	167.3	9.9
	1	1786.8	230.9	202.9	37.6
	2	1789.6	98.9	207.1	19.6
	5	2299.5	111.2	397.9	30.8
	10	2514.4	69.6	1166.6	163.9
HPMC	0.5	73.5	28.3	16.2	6.3
	1	145.6	62.3	28.7	17.8
	2	325.7	137.1	60.7	16.1
	5	607.8	358.7	180.7	64.5
	10	779.4	265.5	527.1	170.0

**Table 3:** Release exponent (*n*), kinetic constant (*k*) and *R*^2^ values of curves fitted on the drug release curves of formulations 2, 3, and 6.

Formulation No.	*n*	*k*	*R* ^2^
2	0.6237	1555	0.9802
3	0.4567	995.6	0.9926
6	0.6671	1360.6	0.9880
